# Inhibition of Methylglyoxal-Mediated Protein Modification in Glyoxalase I Overexpressing Mouse Lenses

**DOI:** 10.1155/2010/274317

**Published:** 2010-07-05

**Authors:** Mahesha H. Gangadhariah, Maneesh Mailankot, Lixing Reneker, Ram H. Nagaraj

**Affiliations:** ^1^Department of Ophthalmology & Visual Sciences, Case Western Reserve University, Cleveland, OH 44106, USA; ^2^Mason Eye Institute, University of Missouri, Columbia, MO 65212, USA

## Abstract

*Objective*. Here we tested the role of Glo I in the prevention of advanced glycation end product (AGE) formation in transgenic mouse lenses. *Methods*. A transgenic animal line that expressed high levels of human Glo I in the lens was developed from the C57B6 mouse strain. The role of Glo I in the inhibition of MGO-AGE formation was tested in organ-cultured lenses. *Results*. Organ culture of Wt and Glo I lenses with 5 mM D, L-glyceraldehyde (GLD) enhanced MGO by 29-fold and 17-fold in Wt lenses and Glo I lenses, respectively. Argpyrimidine levels were 192 ± 73 pmoles/mg protein, and hydroimidazolone levels were 22 ± 0.7 units/*μ*g protein in GLD-incubated Wt lenses. In Glo I lenses, formation of AGEs was significantly inhibited; the argpyrimidine levels were 82 ± 18 pmoles/mg protein, and the HI levels were 2.6 ± 2.3 units/*μ*g protein. Incubation of Wt lens proteins with 5 mM ribose for 7 days resulted in the formation of pentosidine. However, the levels were substantially higher in Glo I lens proteins incubated with ribose. *Conclusion*. Our study provides direct evidence that Glo I activity plays an important role in the regulation of AGE synthesis in the lens; while Glo I activity blocks the formation of MGO-AGEs, it might promote the formation of sugar-derived AGEs.

## 1. Introduction

Lens proteins undergo numerous physicochemical changes during aging and cataract formation. Some of the prominent changes are protein crosslinking, chromophore and fluorophore adduct formation on proteins, deamidation and truncation [1]. Several mechanisms have been proposed for such changes, including the Maillard reaction. The Maillard reaction is a nonenzymatic reaction of reactive carbonyls, such as glucose and ascorbate oxidation products, with the amino groups of proteins. The reaction proceeds through the formation of an Amadori product which, by a series of reactions, produces advanced glycation end products (AGEs) on proteins [2]. Many AGEs have been detected in the human lens, that include, glucosepane [3], pentosidine [4], *N* ^*ε*^-carboxymethyllysine [5], pyrraline [6], K2P [7], and vesperlysine [8]. While vesperlysine and K2P are lysine-lysine crosslinking adducts, pentosidine and glucosepane are lysine-arginine adducts. These AGEs progressively accumulate in aging lenses and accumulate at a higher rate in cataractous lenses. 

Methylglyoxal (MGO) is an additional AGE precursor in the lens. It is an *α*-dicarbonyl compound produced nonenzymatically from the triose phosphate intermediates of glycolysis [9]. MGO reacts rapidly with arginine residues on proteins to form hydroimidazolone (HI, there are three isomers of this product) and argpyrimidine adducts [10]. While argpyrimidine is a blue fluorescent product, HI isomers are nonfluorescent and nonchromophoric adducts. MGO also reacts with the lysine residues of proteins to produce *N* ^*ε*^-carboxyethyl lysine [5], MOLD, and MODIC [3, 11]. MOLD is an imidazolium salt that is formed from a crosslinking adduct between two lysine residues, and MODIC is a lysine-arginine crosslinking structure. These AGEs are present in relatively high concentrations in aged and cataractous human lenses [11–13]. 

MGO is metabolized by glyoxalase and aldo-keto reductases in the lens. However, glyoxalase constitutes the major route of metabolism [14]. Glyoxalase is comprised of two enzymes, glyoxalase I (Glo I), which converts hemithioacetal (formed nonenzymatically from the reaction of glutathione and MGO) to S-D-lactoyl glutathione, and glyoxalase II (Glo II), which catalyzes the conversion of S-D-lactoyl glutathione to D-lactate [15]. 

Glo I appears to be critical for reducing MGO concentrations and subsequent AGE formation in micro- and macrovascular endothelial cells [16, 17] and for the survival of human retinal capillary pericytes in high glucose environments [18]. Its activity in the lens is enhanced during diabetes, possibly as a means to cope with increased MGO concentrations [19]. In the rat lens, Glo I inhibition led to an increase in MGO and AGE content [20]. These findings suggest that Glo I is critical for the reduction of MGO accumulation and AGE formation. To gain further insight into the role of Glo I in the lens, we developed a transgenic mouse line that specifically overexpresses human Glo I in lens epithelial and fiber cells. Using lenses from this animal model, we show that Glo I inhibits MGO-derived AGE formation. Surprisingly, enhanced Glo I activity led to higher levels of a sugar-derived AGE, pentosidine, in ribose incubated-lens proteins when compared to the levels observed for Wt lens proteins. The findings in this study clearly implicate Glo I in the prevention of MGO-mediated AGE synthesis but at the same time suggest that Glo I may enhance sugar-mediated AGE synthesis in the lens.

## 2. Materials and Methods

M-199 medium with Earle's salts, reduced glutathione (GSH), 5,5′-dithiobis 2-nitro-benzoic acid (DTNB), ethylenediaminetetraacetic acid (EDTA), DL-glyceraldehyde, heptafluorobutyric acid, 6-hydroxy-2, 4, 5-triaminopyrimidine (TRI), HEPES, trichloroacetic acid (TCA), sodium carbonate, streptozotocin, and phenylmethyl sulfonyl fluoride (PMSF) were purchased from Sigma Chemical Co, St. Louis, MO. HI monoclonal antibody was made in mice by immunizing with HI coupled to KLH (Kanade et al., unpublished).

## 3. Generation of Glyoxalase I Transgenic Animals

**Figure 1:**
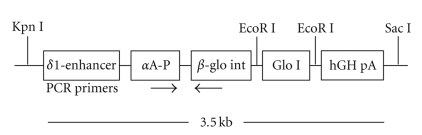
Human Glo I minigene construct. The human Glo I gene was linked to the chimeric promoter that contains the mouse *α*A-crystallin promoter (*α*A-P) and chick *δ*1-crystallin lens enhancer (*δ* -en), which drives the expression of Glo I specifically in the lens epithelium and fiber cells.

The studies complied with the ARVO Statement on the Use of Animals in Ophthalmic and Vision Research and were approved by the Case Western Reserve University Institutional Animal Care and Use Committee. A transgenic mouse line was produced by the standard pronuclear microinjection technique. The details of the transgene DNA construct are illustrated in Figure 1. The human glyoxalase I (Glo I) gene was inserted between EcoR1 sites of a minigene construct that contained a chick *δ*1-crystallin lens enhancer upstream of the *α*A-crystallin promoter and a rabbit *β*-globin intron. Human growth hormone polyA was inserted downstream from the *α*A-crystallin promoter. At 2 to 3 weeks after birth, tail biopsies were obtained, and genomic DNA was screened for transgene integration by PCR using the forward primer 5′- TCT  GAG  AGC  CTC  TGC  TGC  TC -3′ and the reverse primer 5′- GGT  CCA  TGG  TGA  TAC  AAG  GGA  C -3′. The identified founders were crossed with wild type C57BL6 to establish a hemizygous line. Homozygous transgenic mouse lines were established by breeding the hemizygous mice within the same line. All experiments were performed using lenses from the homozygous line.

## 4. In Situ Hybridization

In situ hybridization was performed with a ^35^S-labeled riboprobe homologous to the human growth hormone (hGH) sequences as described previously in [21].

## 5. Morphological Changes

Immediately after dissection, the eyes were fixed in 10% neutral-buffered formalin, embedded in paraffin, cut into 5-*μ*m sections, and stained with hematoxylin and eosin using the standard procedure. After rehydration in xylene and ethanol series, paraffin-embedded eye sections were treated with citrate buffer (pH 6.0) for 20 min at 70°C, cooled and then incubated in 3.0% hydrogen peroxide to block endogenous peroxidase. The sections were incubated with streptavidin D and biotin blocking solution for 15 min each at room temperature and then in mouse-on-mouse (M.O.M, Vector Laboratories, CA) Ig blocking solution for 1 hr at room temperature. After washing in PBS, the sections were incubated in M.O.M diluent, followed by incubation in mouse anti-Glo I mAb [22] diluted to 24 *μ*g/ml in PBS overnight at 4°C. After being thoroughly washed in PBS, the slides were incubated with M.O.M biotinylated antimouse IgG reagent and rinsed thoroughly in PBS. The slides were then incubated in ABC Vectastain Elite Peroxidase (Vector Laboratories) and rinsed in PBS. The sections were stained by incubating in 3, 3′-diaminobenzidine substrate, rinsed thoroughly in deionized water, and counterstained with hematoxylin. The slides were viewed with an Olympus BX-60 upright microscope (Tokyo, Japan). Color images were captured using a SPOT RT Slider camera (Diagnostic Instruments, MI) connected to a Macintosh computer using Spot software version 3.5.5.

## 6. Lens Organ Culture

The mouse lenses from 8-week-old Wt and transgenic animals were dissected out of the eye by a posterior approach without the dissecting tools coming into direct contact with the lens. The lenses were cultured in modified TC-199 media according to Shamsi et al. [20]. Briefly, the lenses were placed in a 24-well plate filled with 2 ml/well of media containing 25 mM HEPES (pH 7.4) and 0.9% sodium carbonate, 30 *μ*g/ml streptomycin and 30 U/ml penicillin. The osmolarity of the media was measured to be ~320 mOsm. The medium was incubated for 2 hr in a 37°C incubator with 5% CO_2_ and 95% air prior to the addition of the lenses. The lenses were incubated for 24 hr, and those that developed haziness were discarded. The lenses were maintained for 48 hr in media containing GLD, and the media was changed after 24 hr. Lenses incubated with media alone served as the control. After the incubation, lenses were washed with 2 ml PBS twice and frozen at −80°C.

## 7. Assay for Glo I Activity

Lenses were homogenized in 0.1 M Tris-HCl buffer, pH 7.4, containing 100 *μ*M PMSF. After homogenization, the extract was centrifuged at 18,000 *g *for 30 min at 4°C. Glo I activity was measured by monitoring the formation of *S*-D-lactoylglutathione at 240 nm over a period of 5 min. The enzyme activity was calculated from the molar extinction coefficient of *S*-D-lactoylglutathione (3370 cm^−1^at 240 nm) and expressed as *μ*moles of *S*-D-lactoylglutathione formed per min per mg of protein.

## 8. MGO Estimation

MGO was estimated according to the method of Espinosa-Mansilla et al. [23]. The lenses were homogenized in 150 *μ*l of 10% TCA and centrifuged. One hundred microliters of supernatant from the TCA extraction was mixed with 1 mM 6-hydroxy-2, 4, 5-triaminopyrimidine (TRI) in 250 *μ*l of sodium acetate buffer at pH 4.05. The mixture was incubated at 60°C for 45  min. The sample was filtered through a 0.45 *μ*m centrifugal filter and injected into a C18 reversed phase HPLC column as per the previously reported procedure in [19]. The MGO content in the samples was calculated by comparison with known quantities of similarly processed MGO standards, and the results were expressed as pmoles/lens. 

## 9. Estimation of GSH

GSH was determined according to Cui and Lou [24]. Each lens was homogenized in 150 *μ*l of 10% TCA, and the homogenate was centrifuged at 10,000 g for 10 min. The supernatant was used for MGO estimation, and the pellet was used for AGE estimation (see below). Twenty microliters of lens TCA supernatant was mixed with 10 *μ*l of DTNB (2 mg/2.5 ml methanol). The volume was adjusted to 200 *μ*l with 1.0 M Tris-HCl buffer, pH 8.2 containing 0.02 M EDTA. The absorbance of the reaction product was measured at 412 nm, and the GSH level was quantified by comparing to GSH standards.

## 10. HPLC Assay for Argpyrimidine and Pentosidine

TCA-pelleted lens protein from the MGO estimation was washed with ether and air dried overnight. The pellet was suspended with 6 N HCl and incubated for 16 hr at 110°C. The acid was evaporated in a Savant SpeedVac system, and the pellet was resuspended in 250 *μ*l of water filtered through a 0.45 *μ*m centrifugal filter. Aliquots of all samples were analyzed by HPLC for argpyrimidine and pentosidine as previously described in [25, 26].

## 11. ELISA for Hydroimidazolone (HI)

Microplate wells were coated overnight with 5 *μ*g of soluble lens protein per well in 50 mM carbonate buffer (pH 9.6) in triplicate. The wells were then washed three times with phosphate-buffered saline-Tween-20 (PBS-T) and incubated with 50 *μ*l of diluted HI monoclonal antibody for 1 hr at 37°C. Following this step, the wells were washed three times with PBS-T and incubated with 50 *μ*l of goat antimouse IgG diluted in PBS-T (1:5000) for 1 hr at 37°C. After the wells were washed with PBS-T, they were incubated with 100 *μ*l of 3, 3′, 5, 5′-tetramethylbenzidine substrate (Sigma). The enzyme reaction was stopped by the addition of 50 *μ*l of 2N H_2_SO_4_, and the absorbance was measured at 450 nm in a Dynex MRX 5000 Microplate Reader. One unit of HI was defined as an increase in 0.01 O.D (at 450 nm)/*μ*g protein.

## 12. Statistical Analysis

Fisher's PLSD test (Statview 5.0; SAS Institute, Inc., Cary, NC) was used to evaluate the differences among treatment groups. We considered *P* ≤ .05 to be statistically significant.

## 13. Results and Discussion

**Figure 2:**
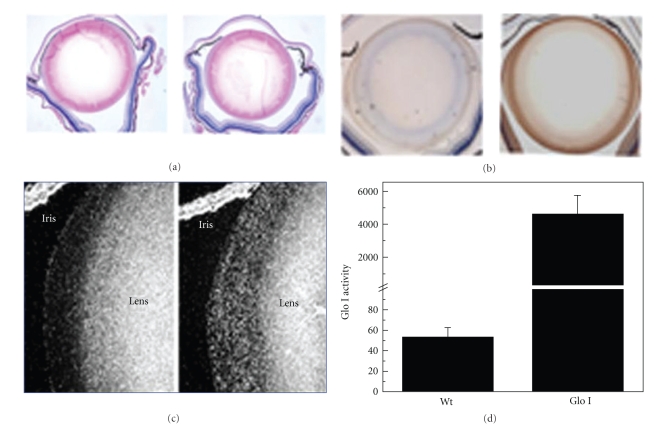
Glo I expression and activity in transgenic mouse lenses. (a) H&E staining of Wt and Glo I transgenic lenses showed no morphological changes in 6-month old animals. No difference was noticeable between the Wt and transgenic lens. (b) Immunohistochemistry using a monoclonal antibody for human Glo I showed human Glo I expression in epithelial cells and outer cortical fiber cells. Glo I immunoreactivity was absent in Wt lenses. (c) In situ hybridization shows Glo I mRNA in outer cortical and epithelial cells in Glo I transgenic animals. (d) Glo I activity was measured in the water-soluble lens proteins. Glo I-catalyzed formation of *S*-D-lactoylglutathione from MGO and GSH was monitored at 240 nm. The data are the mean ± SD from 4 lenses.

Histological examination of lenses from 6-month-old homozygous transgenic animals did not show any morphological changes when compared to the lens of a Wt mouse of a similar age (Figure 2(a)). Immunohistological examination revealed that Glo I was overexpressed both in epithelial cells and in outer cortical fiber cells (Figure 2(b)). Glo I overexpression was further confirmed by in situ hybridization, which showed high levels of Glo I mRNA in epithelial cells and fiber cells (Figure 2(c)). Glo I activity in transgenic lenses was approximately 86-fold higher than in Wt lenses (Figure 2(d)). 

**Figure 3:**
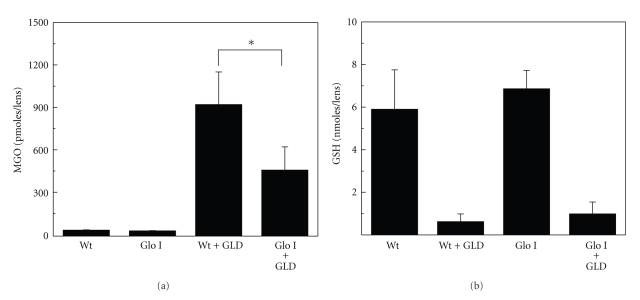
MGO and GSH levels in organ-cultured lenses. Lenses were organ cultured for 48 hr in the absence or presence of 5 mM GLD. Lenses were homogenized in 10% TCA, and supernatants were used for MGO and GSH estimation as described in the Materials and Methods. MGO levels (a) are expressed as pmoles/lens (*P < .005), and GSH levels (b) are expressed as nmoles/lens. The average wet weight of each lens was 5.5 mg. The results are the mean ± SD from 6 lenses.

To determine if the overexpression of Glo I prevents MGO-AGE formation, the lenses were incubated with 5 mM GLD. Direct exposure of the lenses to MGO was avoided, as MGO becomes cytotoxic above 500 *μ*M and causes opacity of the lens. We have previously shown that incubation of lenses with GLD results in high levels of MGO and MGO-AGEs in organ-cultured rat lenses [20]. Incubation of Wt lenses with GLD resulted in a profound accumulation of MGO. This was significantly reduced in Glo I lenses (Figure 3(a)), suggesting that the overexpression of Glo I inhibited MGO accumulation in the lens. These results are in line with the previous reports showing that Glo I overexpression reduced intracellular MGO levels in human umbilical vein endothelial cells [16] and reduced MGO-AGEs in rat renal tubular epithelial cells [27]. In addition, it has been shown that a reduction of Glo I activity results in MGO-AGEs in cells [17, 28], which is compatible with the present findings.

GSH is a cofactor of Glo I. Our previous study has shown that incubation with GLD reduces GSH levels in rat lenses, possibly because of enhanced oxidation [20]. We investigated whether GLD also reduced GSH in mouse lenses. Upon incubation with GLD, GSH levels were reduced nearly 10-fold in both Wt and Glo I lenses (Figure 3(b)). Even though the residual GSH levels were far smaller than those present in lenses incubated without GLD, the levels found in lenses incubated with GLD could have been sufficient to support Glo I activity. This assertion is supported by the fact that MGO levels in GLD-incubated Glo I lenses were approximately 50% lower than those present in GLD-treated Wt lenses (Figure 3(a)). These results are in agreement with our previous studies with rat lenses where we found that GSH levels, even though precipitously diminished upon incubation with GLD, were sufficient to reduce the levels of an MGO-AGE in the lens [20].

**Figure 4:**
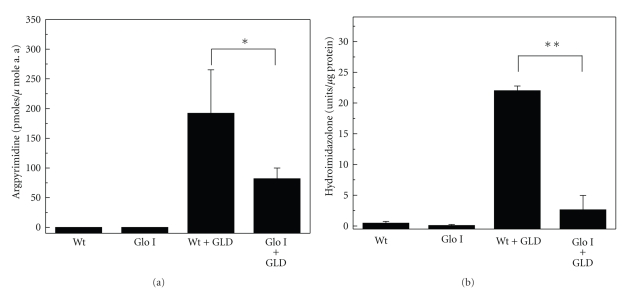
MGO-derived AGEs are inhibited by Glo I overexpression. (a) Argpyrimidine content in lenses cultured in the presence of GLD. TCA precipitated lens protein was hydrolyzed by 6 N HCl and subjected to HPLC analyses as described in the Materials and Methods. The results are the mean ± SD of 6 lenses. (*P < .001). (b) HI estimation by ELISA. Microplate wells were coated overnight with 5 *μ*g of soluble lens protein per well and incubated with a monoclonal antibody for HI followed by goat antimouse IgG. The results are the mean ± SD from 4 lenses. (**P < .0001).

The MGO-AGE levels were reduced as a consequence of the increased Glo I activity and decreased MGO levels. Incubation of the lenses with GLD resulted in nearly 200 pmoles/*μ*mole amino acid argpyrimidine in Wt lenses. This was reduced by nearly 2-fold (*P* < .001) in GLD-treated Glo I lenses (Figure 4(a)). Similarly, the HI content was reduced nearly 10-fold (*P* < .0001) in Glo I lenses when compared to Wt lenses (Figure 4(b)). These results suggest that Glo I regulates MGO-AGE formation in the lens. Our results are compatible with previous studies that have shown similar effects of Glo I overexpression on the reduction of MGO-AGEs [16, 27]. 

**Figure 5:**
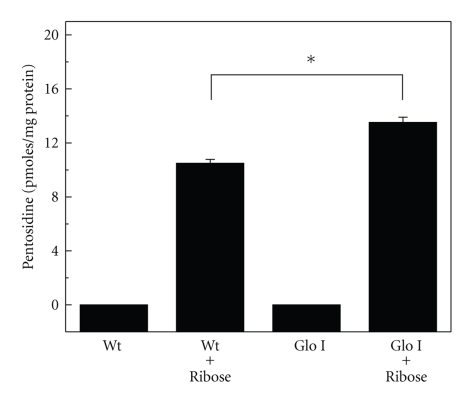
Promotion of pentosidine synthesis by Glo I overexpression. Wt and Glo I lens homogenates (5 mg/ml) were incubated with 5 mM ribose for 7 days and analyzed by HPLC for pentosidine. The results are the mean ± SD from 3 experiments. (*P < .0001).

Pentosidine is an AGE that is formed as a crosslinking adduct between a lysine and an arginine residue in proteins [29]. We have previously shown that MGO inhibits pentosidine synthesis from ribose and ascorbate [26]. We reasoned that MGO occupies arginine residues because of its extreme reactivity with it and thereby blocks pentosidine formation. To test whether Glo I overexpression prevented MGO-mediated inhibition of pentosidine synthesis, we incubated water soluble proteins (without dialysis, to preserve GSH) from Wt and Glo I lenses with 5 mM ribose for 7 days at pH 7.4. While the incubation of ribose with Wt lens proteins resulted in the accumulation of pentosidine, as expected, incubation of ribose with Glo I lens proteins resulted in even higher concentrations (30% more) of pentosidine (Figure 5). This suggests that MGO is generated during ribose-mediated glycation and that MGO is metabolized by Glo I in isolated lens proteins. In fact, in a previous study, we have shown that MGO-mediated argpyrimidine synthesis occurs during glycation by ribose [30], implying that MGO is produced during the reaction. Thus, the present study confirms those results and provides a basis for the argument that Glo I overexpression may not be the best strategy to reduce AGEs in cells and tissues.

## 14. Conclusions

In conclusion, our study provides direct evidence for the modulation of AGE synthesis by Glo I. Determining whether MGO-mediated AGE inhibition is beneficial or harmful requires further work, as our studies have shown that mild modification of lens *α*A-crystallin makes it a better chaperone protein [31, 32]. The chaperone property of *α*A-crystallin has been proposed to play an important role in maintaining the transparency of the aging lens. The other finding that Glo I-mediated removal of MGO could promote synthesis of AGEs from sugars suggests that overexpression of Glo 1 to deplete MGO may be counterintuitive for prevention of AGE synthesis in the lens.

## Abbreviations

AGEs: advanced glycation end products
TCA: trichloroacetic acid
HI: HI
DTNB: 5, 5′-dithiobis (2-nitro-benzoic acid)
GLD: DL-glyceraldehyde
GSH: glutathione reduced form
MGO: methylglyoxal
TRI: 6-hydroxy-2,4,5-triaminopyrimidine
HFBA: heptafluorobutyric acid
DAB: 3,3′-diaminobenizidine.

## References

[1] Bron AJ, Vrensen GFJM, Koretz J, Maraini G, Harding JJ (2000). The ageing lens. *Ophthalmologica*.

[2] Monnier V, Sell D, Masoro EJ (1995). Aging of long-lived proteins: extracellular matrix (collagens, elastins, proteoglycans) and lens crystallins. *Handbook of Physiology*.

[3] Bieme KM, Fried DA, Lederer MO (2002). Identification and quantification of major Maillard cross-links in human serum albumin and lens protein: evidence for glucosepane as the dominant compound. *Journal of Biological Chemistry*.

[4] Nagaraj RH, Sell DR, Prabhakaram M, Ortwerth BJ, Monnier VM (1991). High correlation between pentosidine protein crosslinks and pigmentation implicates ascorbate oxidation in human lens senescence and cataractogenesis. *Proceedings of the National Academy of Sciences of the United States of America*.

[5] Ahmed MU, Brinkmann Frye E, Degenhardt TP, Thorpe SR, Baynes JW (1997). N*ε*-(carboxyethyl)lysine, a product of the chemical modification of proteins by methylglyoxal, increases with age in human lens proteins. *Biochemical Journal*.

[6] Nagaraj RH, Sady C (1996). The presence of a glucose-derived Maillard reaction product in the human lens. *FEBS Letters*.

[7] Cheng R, Feng Q, Argirov OK, Ortwerth BJ (2004). Structure elucidation of a novel yellow chromophore from human lens protein. *Journal of Biological Chemistry*.

[8] Tessier F, Obrenovich M, Monnier VM (1999). Structure and mechanism of formation of human lens fluorophore LM-1. Relationship to vesperlysine A and the advanced Maillard reaction in aging, diabetes, and cataractogenesis. *Journal of Biological Chemistry*.

[9] Thornalley PJ (1996). Pharmacology of methylglyoxal: formation, modification of proteins and nucleic acids, and enzymatic detoxification—a role in pathogenesis and antiproliferative chemotherapy. *General Pharmacology*.

[10] Lo TWC, Westwood ME, McLellan AC, Selwood T, Thornalley PJ (1994). Binding and modification of proteins by methylglyoxal under physiological conditions: a kinetic and mechanistic study with *N*
*α*-acetylarginine, *N*
*α*-acetylcysteine, and *N*
*α*-acetyllysine, and bovine serum albumin. *Journal of Biological Chemistry*.

[11] Chellan P, Nagaraj RH (1999). Protein crosslinking by the Maillard reaction: dicarbonyl-derived imidazolium crosslinks in aging and diabetes. *Archives of Biochemistry and Biophysics*.

[12] Padayatti PS, Ng AS, Ucbida K, Glomb MA, Nagaraj RH (2001). Argpyrimidine, a blue fluorophore in human lens proteins: high levels in brunescent cataractous lenses. *Investigative Ophthalmology and Visual Science*.

[13] Ahmed N, Thornalley PJ, Dawczynski J (2003). Methylglyoxal-derived HI advanced glycation end-products of human lens proteins. *Investigative Ophthalmology and Visual Science*.

[14] Vander Jagt DL, Hassebrook RK, Hunsaker LA, Brown WM, Royer RE (2001). Metabolism of the 2-oxoaldehyde methylglyoxal by aldose reductase and by glyoxalase-I: roles for glutathione in both enzymes and implications for diabetic complications. *Chemico-Biological Interactions*.

[15] Thornalley PJ (1993). The glyoxalase system in health and disease. *Molecular Aspects of Medicine*.

[16] Shinohara M, Thornalley PJ, Giardino I (1998). Overexpression of glyoxalase-I in bovine endothelial cells inhibits intracellular advanced glycation endproduct formation and prevents hyperglycemia-induced increases in macromolecular endocytosis. *Journal of Clinical Investigation*.

[17] Padayatti PS, Jiang C, Glomb MA, Uchida K, Nagaraj RH (2001). High concentrations of glucose induce synthesis of argpyrimidine in retinal endothelial cells. *Current Eye Research*.

[18] Miller AG, Smith DG, Bhat M, Nagaraj RH (2006). Glyoxalase I is critical for human retinal capillary pericyte survival under hyperglycemic conditions. *Journal of Biological Chemistry*.

[19] Staniszewska MM, Nagaraj RH (2006). Upregulation of glyoxalase I fails to normalize methylglyoxal levels: a possible mechanism for biochemical changes in diabetic mouse lenses. *Molecular and Cellular Biochemistry*.

[20] Shamsi FA, Sharkey E, Creighton D, Nagaraj RH (2000). Maillard reactions in lens proteins: methylglyoxal-mediated modifications in the rat lens. *Experimental Eye Research*.

[21] Reneker LW, Chen Q, Bloch A, Xie L, Schuster G, Overbeek PA (2004). Chick *δ*1-crystallin enhancer influences mouse *α*A-crystallin promoter activity in transgenic mice. *Investigative Ophthalmology and Visual Science*.

[22] Mailankot M, Padmanabha S, Pasupuleti N, Major D, Howell S, Nagaraj RH (2009). Glyoxalase I activity and immunoreactivity in the aging human lens. *Biogerontology*.

[23] Espinosa-Mansilla A, Durán-Merás I, Salinas F (1998). High-performance liquid chromatographic-fluorometric determination of glyoxal, methylglyoxal, and diacetyl in urine by prederivatization to pteridinic rings. *Analytical Biochemistry*.

[24] Cui X-L, Lou MF (1993). The effect and recovery of long-term H_2_O_2_ exposure on lenss morphology and biochemistry. *Experimental Eye Research*.

[25] Wilker SC, Chellan P, Arnold BM, Nagaraj RH (2001). Chromatographic quantification of argpyrimidine, a methylglyoxal-derived product in tissue proteins: comparison with pentosidine. *Analytical Biochemistry*.

[26] Puttaiah S, Biswas A, Staniszewska M, Nagaraj RH (2007). Methylglyoxal inhibits glycation-mediated loss in chaperone function and synthesis of pentosidine in *α*-crystallin. *Experimental Eye Research*.

[27] Kumagai T, Nangaku M, Kojima I (2009). Glyoxalase I overexpression ameliorates renal ischemia-reperfusion injury in rats. *American Journal of Physiology*.

[28] Morcos M, Du X, Pfisterer F (2008). Glyoxalase-1 prevents mitochondrial protein modification and enhances lifespan in *Caenorhabditis elegans*. *Aging Cell*.

[29] Sell DR, Monnir VM (1989). Structure elucidation of a senescence cross-link from human extracellular matrix. Implication of pentoses in the aging process. *Journal of Biological Chemistry*.

[30] Shipanova IN, Glomb MA, Nagaraj RH (1997). Protein modification by methylglyoxal: chemical nature and synthetic mechanism of a major fluorescent adduct. *Archives of Biochemistry and Biophysics*.

[31] Nagaraj RH, Oya-Ito T, Padayatti PS (2003). Enhancement of chaperone function of *α*-crystallin by methylglyoxal modification. *Biochemistry*.

[32] Biswas A, Lewis S, Wang B (2008). Chemical modulation of the chaperone function of human *α*A-crystallin. *Journal of Biochemistry*.

